# Glicogenose Tipo I (Doença de Von Gierke): Relato de Dois Casos com Grave Dislipidemia

**DOI:** 10.36660/abc.20190037

**Published:** 2020-05-11

**Authors:** Julia Maria Avelino Ballavenuto, Jéssica D´Ório Dantas de Oliveira, Renato Jorge Alves

**Affiliations:** 1 Santa Casa de Misericórdia de São Paulo São PauloSP Brasil Irmandade da Santa Casa de Misericórdia de São Paulo, São Paulo, SP - Brasil

**Keywords:** Doença de Depósito de Glicogênio Tipo 1/complicações, Gluconeogênese, Dislipidemias, Hepatomegalia, Hipoglicemia, Glucose-6-Fosfatase

## Introdução

São denominadas glicogenoses as afecções decorrentes do erro do metabolismo hereditário, com consequente anormalidade na concentração e/ou na estrutura do glicogênio em qualquer tecido do organismo. Atualmente, existem 14 tipos de doenças do armazenamento de glicogênio, que são classificados de acordo com a deficiência enzimática ou transportadora e a distribuição desses defeitos em diferentes órgãos.^1^

Em 1929, Edgar von Gierke descreveu aumento da concentração de glicogênio em tecidos de autópsias de jovens com manifestações hemorrágicas. Já em 1952, Gerty & Cori analisaram biópsias hepáticas de pacientes com sintomas semelhantes, com a constatação de ausência parcial ou total da enzima glicose-6-fosfatase (G6Pase) – denominando então a Doença de von Gierke. Nordlie et al., em estudos durante a década de 1970, também a partir de biópsias hepáticas, observaram níveis normais da enzima G6Pase, porém com sua atividade diminuída.^2^ Dessa forma, a glicogenose tipo I é caracterizada pela deficiência da G6Pase, enzima-chave no metabolismo do glicogênio, o que leva à redução na glicogenólise e gliconeogênese e, consequentemente, ao acúmulo hepático de glicose-6-fosfato (G6P).^2^

A G6Pase é composta por uma unidade catalítica e por três translocases. Podemos ainda agrupar a glicogenose tipo I em subtipos, de acordo com qual sítio enzimático é deficiente: subtipo Ia (G6P-Ia), que corresponde à deficiência na unidade catalítica; subtipos Ib, Ic e Id, que se referem à deficiência das translocases 1, 2 e 3, respectivamente. Também foi demonstrada deficiência da subunidade catalítica SP, caracterizando, assim, o subtipo IaSP. O diagnóstico dos subtipos é confirmado por biópsia hepática, com determinação da atividade da enzima G6Pase na amostra.^2^

O subtipo Ia possui caráter autossômico recessivo, representando cerca de 80% dos pacientes com deficiência na atividade da G6Pase tipo I, com prevalência de 1:100.000 na população mundial.^3^ Manifesta-se, geralmente, a partir dos 3 a 4 meses de idade, com consequente acúmulo anormal de glicogênio em fígado, rins e intestino, manifestando-se principalmente com hipoglicemia, hiperuricemia, acidose lática e dislipidemia grave. A hipoglicemia geralmente se manifesta na emergência por tremores, convulsões, cianose e apneia e, a longo prazo, pode cursar com retardo no crescimento. No exame físico nota-se hepatomegalia não dolorosa com fígado de bordas lisas palpável abaixo do rebordo costal direito, abdome de aspecto globoso por deposição de gordura abdominal, muitas vezes em um indivíduo de baixa estatura e com fáscies de boneca.^3^ Como complicações tardias, esses pacientes podem apresentar aumento do tamanho renal (com ou sem piora da função renal), adenomas hepáticos (com transformação rara em hepatocarcinoma) e neutropenia (tendências a infecções de repetição).^3 - 5^

A hipertrigliceridemia, mais proeminente no subtipo G6P-Ia, se relaciona à maior morbidade a longo prazo, tendo em vista sua associação com pancreatite e adenomas hepáticos.^6^

Até o início dos anos 2000, eram utilizados rotineiramente estudos enzimáticos efetuados em biópsias de tecido hepático. Atualmente, o diagnóstico é feito com base na análise de mutações nos genes da G6Pase (Glicose-6-fosfatase) e do G6PT (trocador da glicose-6-fosfato/fosfato) por PCR-RFLP (técnica do polimorfismo de comprimento de fragmento), ou por sequenciação direta do gene, associada a manifestações clínicas e laboratoriais. Os estudos enzimáticos ficam reservados aos casos inconclusivos.^7^

Pacientes com transtornos G6P podem apresentar critérios para síndrome metabólica, principalmente hipertrigliceridemia,^8^ níveis baixos de lipoproteínas de alta densidade (HDL) e aumento da circunferência abdominal. Nesse contexto, um monitoramento de doenças cardiovasculares em adultos com G6P seria justificado. Ainda podemos ter pacientes com hipertensão arterial sistêmica (HAS), em menor proporção, geralmente relacionada a alterações renais, que podem surgir a partir da segunda década de vida. Glomeruloesclerose segmentar-focal, nefropatia por gota e nefrocalcinose são as possíveis etiologias da lesão renal. A proteinúria é um achado frequente. No entanto, as alterações renais apresentam boa resposta ao tratamento dietético, justificando o fato de as alterações renais não serem frequentes.^2^

Relatamos duas pacientes com doença do armazenamento, G6P-Ia, associada a dislipidemia grave e de difícil controle, irmãs e filhas de pais consanguíneos (primos de 1º grau), com pai falecido e mãe com tireoidite de Hashimoto, sem relato de outras comorbidades.

Caso 1: Paciente MCS, 24 anos. No primeiro ano de vida, em 1994, foi internada devido a quadro de febre, vômitos, glicosúria, taquipneia, acidose metabólica e hipoglicemia. Naquele momento, foi iniciada investigação do caso, sendo evidenciados altos níveis sanguíneos de colesterol total e triglicérides, além de hiperuricemia e acidose metabólica. Aventada a hipótese de glicogenose hepática tipo I, posteriormente confirmada mediante biópsia hepática e quadro clínico. Foram descartadas outras patologias relacionadas a erros inatos do metabolismo. Exames laboratoriais evidenciaram, naquele momento, hemoglobina glicada de 4,2% e glicemia 65mg/dL. Iniciado o tratamento dietético, com reposição de carboidratos (maisena) desde a infância, apresentando melhora da hipoglicemia. Notava-se elevação de transaminases e, por volta dos 3 anos de idade, apresentou elevações dos níveis de ácido úrico, que evoluíram em queda progressiva com o passar dos anos, até sua normalização na vida adulta. Ao longo da vida, desenvolveu recorrentes episódios de hipoglicemias e infecções, com internação em unidade de terapia intensiva, aos 4 anos, por laringite e broncopneumonia. Sofreu fratura de fêmur aos 9 anos, provavelmente por baixa densidade óssea, quando após realização de nova biópsia hepática, evidenciou-se sinais de fibrose septal. Recebeu o diagnóstico de tireoidite de Hashimoto aos 14 anos (anticorpo anti-tireoperoxidase positivo), com cintilografia de tireoide evidenciando bócio difuso. Mais tarde, aos 18 anos, foram diagnosticados adenomas hepáticos em exames de controle e, aos 22, foi necessária a realização de hepatectomia lateral em segmento II, por adenoma de 4,5cm. Com relação ao desenvolvimento pondero-estatural, a paciente apresentou-se eutrófica, no entanto, apresentando baixa estatura até a vida adulta. Atualmente, apresenta Índice de Massa Corporal (IMC) dentro da faixa de normalidade. Durante a infância e adolescência, apresentou altas concentrações de colesterolemia e trigliceridemia, com pouca melhora após início do tratamento dietético ( Tabela 1 ). A terapêutica farmacológica para dislipidemia foi introduzida aos 20 anos. Apesar da regular adesão ao tratamento, os valores de colesterol total e, principalmente, de triglicérides, mantinham-se constantemente elevados. Desse modo, optou-se por terapêutica com estatina de alta potência e ciprofibrato, com boa tolerância pela paciente, sem desenvolvimento de efeitos colaterais, apresentando melhora parcial dos valores anteriormente encontrados. Durante toda a evolução clínica, não foram observadas alterações renais.

**Tabela 1:** – Dados comparativos dos casos – resultados laboratoriais ao longo dos anos

	CT		LDL-c		HDL-c		TG		AU		Gli		TGO		TGP	

Paciente	1	2	1	2	1	2	1	2	1	2	1	2	1	2	1	2
**1996**	246	*	-	*	48	*	711	*	-	x	65	x	168	x	136	x
**1999**	269	117	-	-	-	-	778	362	9,0	-	-	-	77	100	62	70
**2002**	260	293	-	-	31	59	581	1059	8,5	8,3	-	-	92	121	101	133
**2005**	260	264	-	86	39	43	766	713	3,5	6,8	79	95	166	205	130	240
**2008**	314	326	-	220	53	39	497	335	-	4,6	-	-	43	251	39	252
**2011**	274	423	-	-	53	53	537	1132	4,9	7,4	60	78	48	96	23	109
2014 **	321	304	195	-	55	52	397	701	-	-	92	96	-	61	122	76
2017 **	282	282	165	164	56	48	303	352	-	-	91	-	49	-	29	-

* Paciente do caso 2 não era nascida. ** Introdução de rosuvastatina 40mg e ciprofibrato 100mg/dia. *CT: colesterol total; TG: triglicérides; LDL-c: lipoproteína de baixa densidade; HDL-c: lipoproteía de alta densidade; AU: ácido úrico; Gli: glicemia; HBA1C: glicohemoglobina; TGO: transaminase gutâmico-oxalacetica; TGP: transaminase glutâmico-pirúvica.*

Caso 2: Paciente GCS, 20 anos, irmã diagnosticada com glicogenose tipo Ia, no primeiro ano de vida. Apresentou hepatomegalia (3cm abaixo do rebordo costal direito) ao nascimento, em 1998. Diante do quadro clinico e dos antecedentes familiares, iniciou-se a investigação de glicogenose. Desde o nascimento, além de hepatomegalia, apresentava alterações nas concentrações de colesterol total e triglicérides, hemoglobina glicada, ácido úrico, lactato e transaminases, fato que fortalecia a suspeita de glicogenose. Realizou a primeira biópsia aos 6 meses de idade, ainda com resultado inconclusivo, entretanto, com sinais de esteatose hepática e discreta fibrose. O diagnóstico foi praticamente confirmado aos 3 anos com nova biópsia resultando em hepatopatia crônica em cirrotização, por provável doença do acúmulo de glicogênio. Aos 4 anos, o teste enzimático foi compatível com o diagnóstico de glicogenose tipo I. Outros exames foram realizados, mas apresentaram-se negativos. A paciente manteve níveis alterados de colesterolemia e de trigliceridemia durante toda a infância e adolescência, quando em maio de 2015, aos 17 anos, iniciou estatina de alta potência em associação a ciprofibrato, 100mg/dia. Apesar das medicações, apresentava hiperlipidemia grave, com altas concentrações plasmáticas de triglicérides e colesterol total ( Tabela 1 ). Assim como ocorreu com a paciente do caso 1, as medicações foram bem toleradas e não foram observados efeitos colaterais. Também não houve manifestações de lesão renal. A paciente manteve-se eutrófica com baixa estatura durante seu desenvolvimento, com IMC normal nos dias de hoje.

Para a investigação de doença aterosclerótica subclínica, foi realizada ultrassonografia doppler de carótidas, sem alterações em ambas as pacientes. A eletroforese de lipoproteínas, para investigação do fenótipo de dislipidemia, mostrou acúmulo de pré-betalipoproteínas, correspondente à fração VLDL (lipoproteína de muito baixa densidade), fenótipo tipo IV de Fredricksson ( Tabela 2 ).

**Tabela 2:** – Eletroforese de lipoproteínas das pacientes

Dosagem	Resultados (%)		Referência (%)

	Paciente 2	Paciente 2	
**Alfa lipoproteina**	28,6	17,8	23-46
**Pré-beta lipoproteina**	36	36,8	3-18
**Beta lipoproteína**	35,4	45,4	42-63
**Lipoproteína a - Lp(a)**	0	0	

## Discussão

A G6P Ia está associada a hipertrigliceridemia e hipercolesterolemia graves, chegando a atingir concentrações plasmáticas de triglicérides até 4.000-6.000mg/dL e de colesterol 400-600 mg/dL.^6^ A hiperlipidemia tem relação com o aumento dos produtos glicolítcos elementares para a síntese do colesterol, como o NADP, NADH, fosfato, glicerol-3-fosfato e coenzima A.^2^ Geralmente, as concentrações de VLDL e de lipoproteínas de baixa densidade (LDL) estão aumentadas, enquanto que as de lipoproteínas de alta densidade (HDL) e as de apolipoproteínas AI, AII estão diminuídas; ainda, as concentrações de apolipoproteínas CIII, B e E encontram-se elevadas. As partículas de VLDL e LDL não são apenas aumentadas em número, como é evidente a partir de níveis aumentados de apolipoproteína B, mas também de tamanho, devido ao acúmulo de triglicérides nessas frações.^6 - 8^

Bernier et al.,9 demonstraram que as prevalências gerais de hipercolesterolemia (31%) e hipertrigliceridemia (67%) são maiores no subtipo Ia que no III. No adulto, as anormalidades bioquímicas tendem a se atenuar, ao contrário da hiperlipidemia, que se mantém na G6P Ia, embora não tenha sido observado maior risco de aterosclerose.^9^

Nos casos relatados, as concentrações de triglicérides eram consideravelmente aumentadas desde a infância, com colesterolemia também elevada, porém em menor proporção, evoluindo assim até a adolescência.

Pode-se questionar se, com o passar da idade, a hipoglicemia tenderia a melhorar como consequência da diminuição da taxa metabólica do organismo e da influência dos hormônios sexuais femininos, além da readaptação da dieta. A eletroforese de lipoproteínas, realizada em nossas pacientes, mostrou aumento da fração pré-beta em ambas. Contudo, na paciente mais jovem, que apresentava perfil lipídico mais alterado, devido à hipertrigliceridemia mais agressiva, detectou-se, também, redução da fração alfa na eletroforese.

O Estudo Europeu sobre a Doença do Armazenamento do Glicogênio Tipo I ( *ESGSD I* ) recomenda seguimento e investigação laboratoriais de rotina (incluindo perfis lipídicos), de acordo com a idade: 0-3 anos a cada 2 meses; 3-20 anos, a cada 3 meses; adultos, a cada 6 meses, bem como monitoramento de doenças cardiovasculares.7 Nesse contexto, a concentração de triglicerídeos é considerada o parâmetro mais útil para o controle metabólico crônico, devido à considerável melhora com a idade na ocorrência de hipoglicemia, nos níveis de lactato sérico e ácido úrico.^10^

Na investigação da doença aterosclerótica subclínica, já que nenhuma das pacientes demonstrou aterosclerose manifesta, realizou-se doppler de carótidas, que não apresentou alterações. No entanto, pacientes saudáveis e de mesma idade, mostraram menor espessura intimal comparadas a portadores de G6Pase.^11^ Ainda com relação à coorte de Bernie et al.,^9^ foi comparada a espessura das camadas íntima e média das artérias carótidas (EIMC) ou *intima-media thickness* ( *IMT* ) e o índice de aumento médio medido pela tonometria da artéria radial, em 28 pacientes com G6P versus 23 no grupo controle. Foi demostrado valor maior de EIMC no grupo G6P em comparação com o grupo controle, p < 0,02 ajustado para a idade, sexo e IMC (índice de massa corporal), além do índice de aumento médio medido pela tonometria da artéria radial, encontrado também maior valor no grupo G6P (16,4% +-14,0%) do que no grupo controle (2,4% +-8,7%) (p <0,001).^8^ Tais dados sugerem que a G6P-Ia pode estar associada a maiores disfunções arteriais e aumento do risco cardiovascular.

Por outro lado, haveria uma possível proteção cardiovascular, com diminuição da adesão plaquetária e, consequentemente, um tempo de sangramento prolongado, proporcionando menor risco de aterotrombose. A detoxicação dos radicais livres parece ser o principal fator de proteção para a integridade da membrana celular, por gerar aumento na produção de NADPH2 e ativação do sistema de detoxicação de radicais livres.^1^

Desde a infância, nossas pacientes apresentavam elevada trigliceridemia, que corresponderia a defeito poligênico, com maior síntese de VLDL, acompanhada ou não de incapacidade de sua metabolização pela lipase lipoproteica.^8^ Posteriormente, na faixa etária dos 10 aos 14 anos, ambas apresentaram elevações proporcionais de colesterolemia e trigliceridemia, habitualmente superiores a 300 mg/dL. Esse perfil lipídico, semelhante ao fenótipo tipo III de Fredrickson, seria ocasionado por alterações na apolipoproteína E e/ou incapacidade de metabolização da IDL (lipoproteína de densidade intermediária).^4^

O perfil lipídico na G6P-Ia geralmente sofre alterações expressivas, principalmente quanto à hipertrigliceridemia, sendo duas das principais complicações a pancreatite e adenomas hepáticos.^7^ Com relação ao tratamento, além de medidas dietéticas específicas, o uso de estatinas e fibratos seria indicado, para melhor controle da dislipidemia, redução do risco cardiovascular e prevenção de episódios de pancreatite.^7^

O manuseio dietético é tradicionalmente baseado na provisão de carboidratos exógenos para compensar a gliconeogenese defeituosa e alcançar a normoglicemia. Dessa forma, são indicadas refeições frequentes, alimentação enteral contínua noturna e a administração de amido de milho não cozido.^12^ O tratamento também abrange o uso de fibratos como forma de prevenção de pancreatite, bicarbonato de sódio e inibidores da xantina oxidase no tratamento da acidose metabólica e da hiperuricemia, respectivamente.^7^ Ambas as pacientes não manifestaram alterações renais até então, o que pode ser atribuído ao tratamento dietético precoce.^2^

Se a hipoglicemia puder ser prevenida, conforme já mencionado, as anormalidades clínicas e bioquímicas, na maioria dos pacientes, tendem a melhorar.^2^ Entretanto, a hiperlipidemia tende a se manter, embora não tenha sido observado maior risco de aterosclerose até o momento. Com sua introdução, o fenótipo dos pacientes com G6P mudou de mortalidade para morbidade, e o foco de atenção se moveu para a prevenção de complicações a longo prazo, tais como as possíveis consequências da dislipidemia grave, dentre outros.^12 - 14^

O manuseio clínico da G6P-Ia ainda precisa de maior entendimento da patologia e, por esse motivo, mais estudos deveriam ser realizados nesse sentido.

## Conclusão

G6P-Ia é uma doença rara e subdiagnosticada, que cursa com dislipidemia grave, além de outras complicações. Graças ao seu diagnóstico precoce e instituição de terapêutica eficaz, é possível melhorar a expectativa de vida desses pacientes.

## References

[1] 1. Wang DQ, Fiske LM, Carreras CT, Weinstein DA. Natural history of hepatocellular adenoma formation in glycogen storage diseases type I. J Pediatr. 2011;159(3):442-6. 10.1016/j.jpeds.2011.02.031 PMC3135733 21481415

[2] 2. Reis CVS, Penna FJ, Oliveira MCC, Roquete MLV. Glicogenose tipo I. J Ped 1999;75(4):277-35. 10.2223/jped.313 14685523

[3] 3. Froissart R, Piraud M, Boudjemline AM, Vianey-Saban C, Petit F, Hubert-Buron A, et al. Glucose-6-phosphatase deficiency. Orphanet J. Rare Dis. 2011;6:27. 10.1186/1750-1172-6-27 PMC3118311 21599942

[4] 4. Vivatrat N, Barshop BA, Jones KL. Severe Hypertriglyceridemia and Recurrent pancreatitis in a girl with Type Ia Glycogen Storage Disease and Type III Hyperlipoproteinemia. Am J Med Genet A. 2009;149A(11):2557-9. 10.1002/ajmg.a.33046 19842193

[5] 5. Marcolongo P, Fulceri R, Gamberucci A, Czegle I, Banhegyi G, Benedetti A. Multiple roles of glucose-6-phosphatases in pathophysiology State of the art and future trends. Biochim et Biophys Acta. 2013; 1830(3):2608-18. 10.1016/j.bbagen.2012.12.013 23266497

[6] 6. Bandsma RH, Smit GP, Kuipers F. Disturbed lipid metabolism in glycogen storage disease type; Eur J Pediatr. 2002;161(Suppl 1):S65-S69. 10.1007/s00431-002-1007-8 12373575

[7] 7. Rake JP, Visser G, Labrune P, Leonard JV, Ulrich K, Smith GPA. Guidelines for management of glycogen disease type I. European Study on Glycogen Storage Disease Type I (ESGSD I). Eur J Pediatric. 2002;161(Suppl 1):S112-S119. 10.1007/s00431-002-1016-7 12373584

[8] 8. Lee PJ, Celermayer DS, Robinson J, Mc Carty SN, Betteridge DJ, Leonard JV. Hyperlipidemia does not impair vascular endothelial function in glycogen storage type Ia. Atherosclerosis. 1994;110(1):95-100. 10.1016/0021-9150(94)90072-8 7857375

[9] 9. Bernier AV, Correia CE, Haller MJ, Theriaque DW, Shuster JJ, Weinstein DA. Vascular dysfunction in glycogen storage disease type I. J Pediatr. 2009; 154(4):588–91. 10.1016/j.jpeds.2008.10.048 PMC3607442 19101686

[10] 10. Derks TG, van Rijn M. Lipids in hepatic glycogen storage diseases: pathophysiology, monitoring of dietary management and future directions. 10. J Inherit 10. Metab10. Dis. 2015;38(3):537-43. 10.1007/s10545-015-9811-2 PMC4432100 25633903

[11] 11. Yekeler E, Dursun M, Emeksiz E, Akkoyunlu M, Akyol Y, Demir F, et al. ctremature atherosclerosis by endothelial dysfunction and increased intima-mediathickness in glycogen storage disease types Ia and III. 11. Turk J 11. Pediatr11. 2007;49(2):115-9. 17907509

[12] 12. Fernandes J, Smit Gpa, Berger R. Outcome of the treatment of glycogen storage disease. Acta Paediatr Jpn.1998;30:57-61. 10.1111/j.1442-200x.1988.tb02537.x 3150236

[13] 13. Kelsch RC, Oliver WJ. Studies on dietary correction of metabolic abnormalities in hepatorenal glycogenosis. Pediatr Res. 1969;3(2):160-70. 10.1203/00006450-196903000-00008 5253123

[14] 14. Moses SW. Historical highlights and unresolved problems in glycogen storage disease type I. Eur J Pediatr. 2002;161(Suppl 1):S2-S9. 10.1007/s00431-002-0997-6 12373565

