# Supplementary material for: Glicogenose Tipo I (Doença de Von Gierke): Relato de Dois Casos com Grave Dislipidemia
Source: Arq Bras Cardiol. 2020 May 11;114(4 Suppl 1):23–6. [Article in Portuguese] doi: 10.36660/abc.20190037 (PMC8149108; doi:10.36660/abc.20190037)
Supplement: Translation

# Translation: Glycogen Storage Disease Type I (Von Gierke disease): Report of Two Cases with Severe Dyslipidemia

**Authors:** Julia Maria Avelino Ballavenuto, Jéssica D´Ório Dantas de Oliveira, Renato Jorge Alves

**Affiliations:** 1 Brazil Irmandade da Santa Casa de Misericórdia de São Paulo, São Paulo, SP - Brazil

**Keywords:** Glycogen Strage Disease Type 1/complications, Gluconeogenesis, Dyslipidemias, Hepatomegaly, Hypoglycemia, Glucose-6-Phosphate

## Introduction

Glycogen storage diseases are a group of disorders caused by inherited errors of metabolism, resulting in abnormal glycogen concentration and/or structure in various body tissues. Nowadays, there are 14 types of glycogen storage disease, which are classified according to enzyme or transporter deficiency and to the different organ distribution of these defects.^1^

In 1929, Edgar von Gierke described an increased deposition of glycogen in body tissues in the autopsy reports of young individuals with hemorrhagic manifestations. In 1952, Gerty and Cori analyzed liver biopsies of patients with similar symptoms, and observed a partial or total absence of glucose-6-phosphatase (G6Pase) enzyme activity – this entity became known as Von Gierke disease. Nordlie et al., in studies carried out in the 70’s, also using liver biopsies, observed normal levels of G6Pase enzyme, but with decreased activity.^2^ Thus, Glycogen Storage Disease Type I is characterized by G6Pase deficiency, a key enzyme in glycogen metabolism, which leads to the reduction in glycogenolysis and gluconeogenesis and, consequently, to hepatic accumulation of glucose-6-phosphate (G6P).^2^

G6Pase is composed of a catalytic subunit and three translocases. GSDI is further divided into subtypes, depending on which enzyme is affected: subtype Ia (GSDIa), which corresponds to a deficiency in the catalytic unit; subtypes Ib, Ic and Id, which refer to deficiency of translocases 1, 2 and 3, respectively. Deficiency in the SP catalytic subunit has also been demonstrated, characterizing, thus, the subtype 1aSP. The diagnosis of the subtypes is confirmed by liver biopsy, with G6Pase activity being determined in tissue samples.^2^

GSDIa is inherited as an autosomal recessive trait, representing about 80% of GSDI patients, with an incidence of 1/100,000 births worldwide.^3^ It commonly manifests between the ages of 3 to 4 months, as a result of abnormal accumulation of glycogen in the liver, kidneys and intestine, by symptoms of hypoglycemia, hyperuricemia, lactic acidemia and severe dyslipidemia. Hypoglycemia usually manifests first as tremors, seizures, cyanosis and apnea and, in the long term, evolves to growth retardation. Physical examination shows non-painful hepatomegaly, with a smooth, palpable liver edge below the right costal margin, globe-like abdomen due to deposition of abdominal fat, often associated with short stature and a doll-like face.^3^ As late complications, these patients may present an increase in kidney size (with or without worsening renal function), hepatic adenomas (with rare transformation into HCC) and neutropenia (a tendency to recurrent infections).^3 - 5^

Hypertriglyceridemia, most prominent in GSDIa, is associated with long-term outcome morbidity, due to its relation with pancreatitis and hepatic adenomas.^6^

Until the early 2000’s, liver biopsies were routinely perfomed to obtain tissue for enzyme analysis. Nowadays, the diagnosis is made based on mutation analysis of the G6Pase (glucose-6-phosphatase) and G6PT (glucose-6-phosphate translocase) genes by PCR-RFLP (Restriction Fragment Length Polymorphism), or by direct gene sequencing, associated with clinical and laboratory manifestations. Enzymatic studies are carried out if results remain inconclusive.^7^

Patients with G6PD mutations may have the criteria for metabolic syndrome, especially hypertriglyceridemia,^8^ reduced levels of high-density lipoproteins (HDL) and increased waist circumference. In this context, the monitoring of cardiovascular diseases in adult patients with GSDI would be justified. To a lesser extent, these patients may present with SAH (systemic arterial hypertension) as well, usually related to renal alterations, which may emerge from the second decade of life on. Focal segmental glomerulosclerosis, gout nephropathy and nephrocalcinosis are the likely etiologies of renal injury. Proteinuria is a frequent finding. However, renal alterations respond positively to dietary treatment, which explains why they are not common.^2^

We report the cases of two patients with GSDIa, associated with severe and difficult to manage dyslipidemia, sisters and daughters of consanguineous parents (first cousins), with deceased father and mother with Hashimoto’s thyroiditis, without reports of other comorbidities.

Case 1: Patient MCS, 24 years of age. During her first year of life, in 1994, she was hospitalized with a picture of fever, vomiting, glycosuria, tachypnea, metabolic acidosis and hypoglycemia. At that moment, an investigation of the case was initiated, which revealed high levels of total cholesterol and triglycerides, in addition to hyperuricemia and metabolic acidosis. The possibility of Glycogen Storage Disease type I was considered, being subsequently confirmed by liver biopsy and the clinical picture. Other pathologies related to inborn errors of metabolism were ruled out. At that time, laboratory tests showed: hemoglobin A1c of 4.2% and blood glucose 65mg/dL. Dietary treatment, with carbohydrate replacement (cornstarch), was initiated in childhood, leading to improvements of hypoglycemia. An increase in transaminases was noted and, around 3 years of age, she presented with hyperuricemia, which evolved with progressive reduction throughout the years, until normalization in adulthood. Throughout her life, she had recurrent episodes of hypoglicemia and infections, with admission to intensive care unit, at the age of 4 years, due to laryngitis and bronchopneumonia. She suffered a femur fracture at the age of 9 years, probably due to low bone density, when a new liver biopsy revealed signs of septal fibrosis. At the age of 14 years, she was diagnosed with Hashimoto’s thyroiditis (positive anti-thyroperoxidase antibody), with thyroid scintigraphy showing a diffuse goiter. Later, at the age of 18 years, hepatic adenomas were diagnosed in check-up investigations and, at the age of 22, left lateral liver resection of segments II was required, due to an adenoma measuring 4.5cm. Regarding pondero-statural development, the patient was eutrophic, but having short stature even as an adult. Nowadays, her Body Mass Index (BMI) is within the normal ranges. During childhood and adolescence, she exhibited high concentrations of cholesterol and triglycerides, with little improvement after starting dietary treatment ( Table 1 ). Pharmacological therapy for dyslipidemia was introduced at the age of 20. Despite regular adherence to treatment, total cholesterol and, especially, triglyceride levels, remained consistently increased. Thus, high-potency statin and ciprofibrate therapy was chosen, which was well tolerated, without any side effects, providing a partial improvement in the results found before. Throughout the clinical course, no renal alterations were observed.

**Table 1:** – Data comparison between the two cases – lab results throughout the years

	TGP		LDL-c		HDL-c		TG		AU		Gli		TGO		TGP	

Patient	1	2	1	2	1	2	1	2	1	2	1	2	1	2	1	2
**1996**	246	*	-	*	48	*	711	*	-	x	65	x	168	x	136	x
**1999**	269	117	-	-	-	-	778	362	9.0	-	-	-	77	100	62	70
**2002**	260	293	-	-	31	59	581	1059	8.5	8.3	-	-	92	121	101	133
**2005**	260	264	-	86	39	43	766	713	3.5	6.8	79	95	166	205	130	240
**2008**	314	326	-	220	53	39	497	335	-	4.6	-	-	43	251	39	252
**2011**	274	423	-	-	53	53	537	1132	4.9	7.4	60	78	48	96	23	109
2014 **	321	304	195	-	55	52	397	701	-	-	92	96	-	61	122	76
2017 **	282	282	165	164	56	48	303	352	-	-	91	-	49	-	29	-

* Case 2 patient was not born. ** Initiation of treatment with rosuvastatin (40 mg) and ciprofibrate (100 mg/day). TC: total cholesterol; TG: triglycerides; LDL-c: low-density lipoprotein cholesterol; HDL-c: high-density lipoprotein cholesterol; UA: uric acid; Glu: blood glucose; HbA1c: glycohemoglobin; TGO: glutamic-oxaloacetic transaminase; TGP: glutamic pyruvic transaminase.

Case 2: Patient GCS, 20 years old, with a sister diagnosed with Glycogen Storage Disease Type Ia, in the first year of life. She was found to have a hepatomegaly 3 cm below the right costal margin at birth, in 1998. In face of her clinical manifestations and family history, research for Glycogen Storage Disease was conducted. Since her birth, in addition to hepatomegaly, she presented alterations in the levels of total cholesterol and triglycerides, glycosylated hemoglobin, uric acid, lactate and transaminases, which supported the suspicion of GSD. She underwent her first biopsy at the age of 6 months, still with inconclusive results, but with signs of hepatic steatosis and mild fibrosis. The diagnosis was virtually confirmed by a new biopsy at the age of 3 years, revealing chronic hepatic disease with cirrhosis, probably caused by GSD. At the age of 4 years, the enzyme test was compatible with the diagnosis of Glycogen Storage Disease Type I. Additional tests were performed whose results were negative. The patient remained with altered levels of cholesterol and triglycerides throughout her childhood and adolescence when, in May 2015, at the age of 17 years, she started ciprofibrate (100mg/day) and high-potency statin combination therapy. In spite of the medications, she had severe hyperlipidemia, with elevated levels of total serum cholesterol and triglycerides ( Table 1 ). As with the patient in Case 1, the medications were well tolerated and there were no side effects. There were no manifestations of renal lesion either. The patient remained eutrophic with short stature during her development, and presents normal BMI values nowadays.

In order to research for subclinical atherosclerotic disease, a carotid Doppler ultrasonography study was performed, which showed no alterations in both patients. Lipoprotein electrophoresis, used to define the phenotype of dyslipidemia, showed accumulation of pre-beta-lipoproteins, corresponding to the very-low-density lipoprotein (VLDL) fraction, Fredrickson classification Type IV ( Table 2 ).

**Table 2:** – Lipoprotein electrophoresis of patients

Dosage	Results (%)		Reference (%)

	Patient 2	Paciente 2	
**Alpha lipoprotein**	28.6	17.8	23-46
**Pre-beta lipoprotein**	36	36.8	3-18
**Beta-lipoprotein**	35.4	45.4	42-63
**Lipoprotein (a) (Lp(a))**	0	0	

## Discussion

GSDIa is associated to severe hypertriglyceridemia and hypercholesterolemia, with serum levels of triglycerides reaching 4.000 to 6.000mg/dL, and serum cholesterol values ranging from 400 to 600 mg/dL.^6^ Hyperlipidemia is related to the increased products of glycolytic pathways, which are essential for cholesterol synthesis, such as NADP, NADH, phosphate, glycerol-3-phosphate and coenzyme A.^2^ Usually, the concentrations of very low-density lipoprotein (VLDL) and low-density lipoprotein (LDL) are increased, whereas the concentrations of high-density lipoproteins (HDL) and of apolipoproteins AI, AII are reduced; in addition, the concentrations of apolipoproteins CIII, B and E are elevated. An increase is observed not only in the number of VLDL and LDL particles, as becomes evident from the elevated apoB levels, but also in their size, due to the triglycerides accumulation in these fractions.^6 - 8^

Bernier et al.9 demonstrated that the overall prevalence of hypercholesterolemia (31%) and hypertriglyceridemia (67%) are higher in GSDIa than in GSD-III patients. In adult populations, the biochemical abnormalities tend to attenuate, unlike hyperlipidemia, which persists in GSDIa, although with no higher related risk of atherosclerosis.^9^

In the cases reported, the concentrations of triglycerides were considerably elevated since childhood, with high cholesterolemia as well, but in lower proportions, thus evolving until adolescence.

It is possible to question whether, over time, hypoglycemia would tend to improve due to decreased metabolic rates in the body and to the influence of female sexual hormones, in addition to nutritional readaptation. Lipoprotein electrophoresis, performed in our patients, showed an increase in the pre-beta fraction in both of them. However, in the youngest patient, who had a more altered lipid profile, due to more severe hypertriglyceridemia, the electrophoresis test also exhibited a decrease in the alpha fraction.

The European Study on Glycogen Storage Disease Type I (ESGSD I) recommends follow-up and routine laboratory tests (including lipid profiles), according with the patient’s age: age 0–3 years every 2 months; 3–20 years every 3 months; adults every 6 months, as well as monitoring of cardiovascular diseases.^7^ In this context, triglyceride concentration is considered the most useful parameter for chronic metabolic control with advanced age, in the presence of hypoglycemia, due to considerable improvements in serum levels of lactate and uric acid.^10^

Regarding the research for subclinical atherosclerosis, since none of the patients had manifested atherosclerosis, a carotid Doppler ultrasound was performed, which revealed no alterations. However, healthy patients and of the same age, showed lower intimal thickness when compared to GSDI patients.^11^ In a cohort of 28 patients with GSD I and 23 control subjects, Bernie et al.^9^ compared carotid intima media thickness (cIMT) and mean augmentation index measured by radial artery tonometry. A greater cIMT value was found in the GSD cohort than in the control group, p < 0.02, adjusted for age, sex, and BMI (body mass index), in addition to mean augmentation index measured by radial artery tonometry, which was also higher in the GSD cohort (6.4% ± 14.0%) than in the control group (2.4% ± 8.7%) (p < 0.001).^8^ These data suggest that GSDIa may be associated with major arterial dysfunction and increased risk for cardiovascular disease.

On the other hand, there would be a possible cardiovascular protection, with decreased platelet adherence and, therefore, prolonged bleeding time, leading to lower risk of atherothrombosis. Detoxification of free radicals seems to be the leading protection factor for cellular membrane integrity, because it enhances NADPH2 production and activates the system of free radical detoxification.^1^

Since their childhood, our patients had high triglycerides, which would correspond to a polygenic defect, with greater VLDL synthesis, followed or not by failure to metabolize it by lipoprotein lipase.^8^ Later, in the 10 to 14 age range, both presented a proportional increase in cholesterol and triglycerides levels, usually greater than 300 mg/dL. This lipid profile, similar to Fredrickson phenotype III, would be the result of changes in apo E and/or due to a failure to metabolize IDL (intermediate density lipoproteins).^4^

The GSPIa lipid profile usually suffers expressive changes, especially in relation to hypertriglyceridemia, with pancreatitis and hepatic adenomas being two of the major complications.^7^ Regarding the treatment, in addition to specific dietary measures, the use of statins and fibrates would be indicated, for a better control of dyslipidemia, reduction of cardiovascular risk and prevention of pancreatitis.^7^

Dietary management is traditionally based on the provision of exogenous carbohydrate to compensate for defective gluconeogenesis and achieve normoglicemia. Thus, frequent meals, continuous overnight enteral feeding and the administration of uncooked cornstarch are indicated.^12^ The treatment also includes the use of fibrates as a way to prevent pancreatitis, sodium bicarbonate and xanthine oxidase inhibitors to treat metabolic acidosis and hyperuricemia, respectively.^7^ Both patients have not presented renal alterations so far, which may be attributed to early dietetic treatment.^2^

If hypoglycemia can be prevented, as mentioned before, the clinical and biochemical abnormalities, in most patients, tend to improve.^2^ Nevertheless, hyperlipidemia tends to persist, although no greater risk of atherosclerosis has been observed so far. Since its introduction, the phenotype of G6PD deficient individuals has changed from mortality to morbidity, and the focus of attention has moved to the prevention of long-term complications, such as the possible consequences of severe dyslipidemia, among others.^12 - 14^

The clinical management of GSPIa still requires a better understanding of the pathology and, for this reason, further studies should be performed in this respect.

## Conclusion

GSPIa is a rare and underdiagnosed disease, which evolves with severe dyslipidemia, among other complications. Early diagnosis and the establishment of efficient therapy contribute to increase the life expectancy of these patients.
